# Cell-Free Total Nucleic Acid-Based Genotyping of Aggressive Lymphoma: Comprehensive Analysis of Gene Fusions and Nucleotide Variants by Next-Generation Sequencing

**DOI:** 10.3390/cancers13123032

**Published:** 2021-06-17

**Authors:** Attila Mokánszki, Réka Bicskó, Lajos Gergely, Gábor Méhes

**Affiliations:** 1 Department of Pathology, Faculty of Medicine, University of Debrecen, H-4032 Debrecen, Hungary; mokanszki.attila@med.unideb.hu; 2 Department of Hematology, Faculty of Medicine, University of Debrecen, H-4032 Debrecen, Hungary; bicsko.reka@med.unideb.hu (R.B.); lgergely@med.unideb.hu (L.G.)

**Keywords:** aggressive lymphoma, liquid biopsy, cell-free nucleic acid, gene fusion, mutation analysis, next-generation sequencing (NGS)

## Abstract

**Simple Summary:**

This study aimed to simultaneously demonstrate pathogenic chromosomal translocations and point mutations from both tissue biopsy and peripheral blood (PB) liquid biopsy (LB) samples of aggressive lymphoma patients. Matched samples were analyzed by next-generation sequencing for the same 125 genes. Eight different gene fusions, including the classical *BCL2*, *BCL6*, and *MYC* genes were detected in the corresponding samples with generally good agreement. Besides, mutations of 29 commonly affected genes, such as *BCL2*, *MYD88*, *NOTCH2*, *EZH2*, and *CD79B* could be identified in the matched samples at a rate of 16/24 (66.7%). Our prospective study demonstrates a non-invasive approach to identify frequent gene fusions and variants in aggressive lymphomas. In conclusion, PB LB sampling substantially supports the oncogenetic diagnostics of lymphomas, especially at anatomically critical sites (such as the central nervous system).

**Abstract:**

Chromosomal translocations and pathogenic nucleotide variants both gained special clinical importance in lymphoma diagnostics. Non-invasive genotyping from peripheral blood (PB) circulating free nucleic acid has been effectively used to demonstrate cancer-related nucleotide variants, while gene fusions were not covered in the past. Our prospective study aimed to isolate and quantify PB cell-free total nucleic acid (cfTNA) from patients diagnosed with aggressive lymphoma and to compare with tumor-derived RNA (tdRNA) from the tissue sample of the same patients for both gene fusion and nucleotide variant testing. Matched samples from 24 patients were analyzed by next-generation sequencing following anchored multiplexed polymerase chain reaction (AMP) for 125 gene regions. Eight different gene fusions, including the classical *BCL2*, *BCL6*, and *MYC* genes, were detected in the corresponding tissue biopsy and cfTNA specimens with generally good agreement. Synchronous *BCL2* and *MYC* translocations in double-hit high-grade B-cell lymphomas were obvious from cfTNA. Besides, mutations of 29 commonly affected genes, such as *BCL2*, *MYD88*, *NOTCH2*, *EZH2*, and *CD79B*, could be identified in matched cfTNA, and previously described pathogenic variants were detected in 16/24 cases (66.7%). In 3/24 cases (12.5%), only the PB sample was informative. Our prospective study demonstrates a non-invasive approach to identify frequent gene fusions and variants in aggressive lymphomas. cfTNA was found to be a high-value representative reflecting the complexity of the lymphoma aberration landscape.

## 1. Introduction

The peripheral blood (PB) of cancer patients represents variable amounts of tumor-derived components, including circulating tumor cells (CTCs), cell-free DNA (cfDNA) and cell-free RNAs (cfRNAs) released from tumor foci of any anatomical location. While the frequency of viable tumor cells proved to be limited and highly inconsistent, plasma cfDNA and cfRNA fractions are now considered potential resources for the real-time genetic assessment of the malignant processes. The principle of non-invasive liquid biopsy (LB) has been successfully transferred to clinical diagnostics and the disease monitoring of solid tumors and aggressive lymphomas [1,2,3,4,5,6,7,8]. These studies provided two main insights: variant allele frequencies often correlate with disease status; and surveilling cfDNA might outperform positron emission tomography/computed tomography (PET/CT) scans in terms of sensitivity—a finding that holds great potential for relapse risk assessment—by quantifying minimal residual disease. Cell-free nucleic acids circulate at low quantities in the PB; consequently, identification of genetic aberrations requires high-sensitivity and high-throughput techniques, such as next-generation sequencing (NGS). cfDNA analysis has tremendous clinical potential, especially for patients with lesions that are difficult to access for biopsy sampling (e.g., brain; deep thoracic or abdominal localisations).

Nevertheless, the LB approach has not been investigated in every detail [9]. Traditionally, structural variants such as chromosomal rearrangements resulting in gene fusions are detected by chromosome karyotyping or fluorescence in situ hybridization (FISH), or more recently, by PCR-based methods (*IGH*-*CCND1*, *IGH*-*BCL2*) following reverse transcription from tumor-derived RNA. As the number of actionable targets grows, the demand for rapid testing from any available specimens, such as the PB also increases.

Recent studies on the utility of an RNA NGS-based assay for lymphoma genotyping using cell-free total nucleic acid (cfTNA) and matched tumor-derived RNA (tdRNA) substrate reported the potential to detect multiple clinically relevant fusion transcripts simultaneously to identify genomic translocations [10,11]. Anchored multiplexed PCR (AMP) was found to be particularly effective for gene fusion detection, and fusions can be identified even without prior knowledge of fusion partners or breakpoints. This technique applies primers, specific for the important genes involved in lymphoma progression, which connect upstream or downstream of an exon–intron boundary and which hybridize to the sequencing adapter [10,12]. Primers are designed for the proximal region of exon–exon junctions involved in the fusions; thus, rearrangements that fall outside the transcribed region of the genes will not be covered. Additional coverage is provided for some targets using supplemental primers. 

Our prospective study aimed to demonstrate the utility of the AMP-based NGS technology for cfTNA genotyping in aggressive lymphoma. PB samples were collected and cfTNA was isolated and quantified from newly diagnosed patients. For comparison, tdRNA from tissue samples of the same patients was isolated. Gene fusions and RNA variants from the two matched sample types (cfRNA and tdRNA) were identified and correlated. For this purpose, a gene panel targeting 125 genes commonly involved in lymphoid malignancies (Archer FusionPlex and Miseq platform) was used. 

## 2. Materials and Methods

### 2.1. Study Cases and Samples

Lymphoma patients were diagnosed and treated at the Department of Hematology, the University of Debrecen from the period of November 2019 to November 2020. Major criteria for the selection were 1. clinical/histological aggressive features and 2. parallel samples available from both neoplastic tissue and peripheral blood for nucleic acid isolation. Formaldehyde-fixed paraffin-embedded tissue (FFPE) samples were collected from, altogether, 24 patients diagnosed with nodal diffuse large B-cell lymphoma (DLBCL, 7 cases), non-nodal DLBCL (9 cases), primary central nervous system lymphoma (PCNSL, 3 cases), follicular lymphoma grade 3a (FL, 3 cases), Burkitt-lymphoma (BL, one case) and one high-grade peripheral T-cell lymphoma (PTCL, one case) at the Department of Pathology, University of Debrecen. Follicular lymphomas were all diagnosed as grade 3A, which was considered aggressive-type B-cell lymphoma with an increased cell proliferation rate. No peripheral blood and leukemic involvement were observed in any of the cases. All tissue samples were taken from the primary lymphoma sites at initial diagnosis. PB samples from the same patients were collected right after diagnosis for genetic analysis. Sampling was agreed upon and supported by written consent. All protocols have been approved by the author’s respective Institutional Review Board for human subjects (IRB reference number: 4941/2018). This study was managed according to the Declaration of Helsinki.

### 2.2. Histology and Immunohistochemistry

Hematoxylin and eosin (H&E)-stained slides were analyzed by pathology specialists. Histological evaluation and interpretation were done according to the WHO classification of lymphatic neoplasias [13,14], which covered the immunohistochemical (IHC) analysis of the intrinsic markers CD10 (clone 56C6, 1:200 dilution, Leica Biosystems, Wetzlar, Germany), BCL6 (clone GI191E/A8, ready-to-use, Roche Diagnostics, Basel Switzerland), MUM1 (clone EAU32, ready-to-use, Leica Biosystems, Wetzlar, Germany), MYC (clone Y69, 1:100 dilution, Abcam, Cambridge, UK), and BCL2 (clone 124, 1:200 dilution, Dako, Agilent Technologies Company, Santa Clara, CA, USA). Additional staining for Ki-67 (clone MIB1, 1:200 dilution, Dako, Agilent Technologies Company, Santa Clara, CA, USA) was done to determine the cell proliferation index. For DLBCL cases, the cell of origin (COO) status was assigned employing the Hans classification [15].

### 2.3. Fluorescence In Situ Hybridization

Fluorescence in situ hybridization (FISH) was performed using *MYC*, *BCL2* and *BCL6* break apart probes to detect gene translocation on FFPE samples according to the manufacturer’s protocol (Metasystems, Altlussheim, Germany).

### 2.4. Tumor and Cell-Free Nucleic Acid Isolation

H&E-stained slides were selected for molecular analysis with a >20% tumor percentage. Genomic tdRNA was extracted from FFPE tissues using ReliaPrep FFPE Total RNA Miniprep System (Promega, Madison, WI, USA) according to the manufacturer’s instructions.

Blood samples were taken in EDTA anticoagulant tubes and were centrifuged at 3000× *g* for 10 min. 5 ± 0.1 mL plasma was spun down (16,000 g, 10 min) to eliminate cell residues. cfTNA was extracted from PB plasma into 30 µL elution buffer using QIAamp Circulating Nucleic Acid Kit (Qiagen, Hilden, Germany). cfDNA, tdRNA, and cfRNA concentrations were measured by the Qubit dsDNA HS Assay Kit using a Qubit 4.0 Fluorometer (Thermo Fisher Scientific, Waltham, MA, USA). 

### 2.5. Next-Generation Sequencing (NGS)

For NGS library preparation, the Archer FusionPlex Lymphoma gene panel (Archer DX, Boulder, CO, USA) was used. Anchored primers were applied for the known translocation partners and reverse primers to hybridize with the sequencing adapters to identify breakpoints and partners [10,11]. A total of 100–250 ng of tdRNA or the matched cfTNA was loaded into the assay. After first-strand cDNA synthesis, a quantitative RT-PCR Pre-seq QC was performed to define the yield of intact RNA in the samples [11]. The final libraries were quantified using a KAPA library quantification kit (Roche, Basel, Switzerland), diluted to a final concentration of 4 nM, and pooled by equal molarity. 

For sequencing on the MiSeq System (MiSeq Reagent kit v3, 600 cycles), all libraries were denatured by adding 0.2 nM NaOH and diluted to 40 pM with hybridization buffer from Illumina (San Diego, CA, USA). The final loading concentration was 10 pM libraries and 1% PhiX. Sequencing was conducted according to the MiSeq instruction manual. Captured libraries were sequenced in a multiplexed fashion with a paired-end run to obtain 2 × 150 bp reads with at least 250X depth of coverage. Trimmed fastq files were generated using MiSeq reporter (Illumina, San Diego, CA, USA), which were analyzed with Archer analysis software (version 6.2.; Archer DX, Boulder, CO, USA). For the alignment, the human reference genome GRCh37 (equivalent UCSC version hg19) was built. Molecular barcode (MBC) adapters were used to count unique molecules and characterized sequencer noise, revealing mutations below standard NGS-based detection thresholds. The sequence quality for each sample was assessed and the cutoff was set to 5% (2% in cfRNA samples) variant allele frequency (VAF). Translocations were stated at over a 5-read fusion sequence, with reads comprising at least 10% of the total reads from gene-specific primers. Gene fusion frequency was calculated for fusion transcript reads and the total reads ratio.

The results were described using the latest version of the Human Genome Variation Society nomenclature for either the nucleotide or protein level. Individual gene variants were cross-checked in the COSMIC (Catalogue of Somatic Mutations in Cancer) and ClinVar databases for clinical relevance. We used the gnomAD v.2.1.1 population database to compare the significance of each gene alteration that is included in our Archer NGS analysis system.

## 3. Results

### 3.1. Patients and Samples

**Figure 1:**
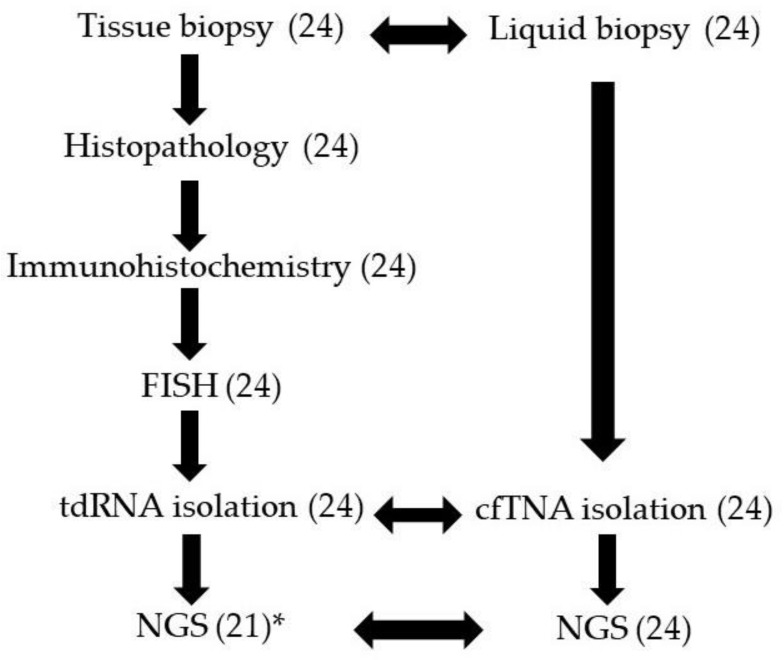
The study workflow of samples from 24 aggressive lymphoma patients. Analyzed sample numbers are indicated in parentheses. *: tdRNA yields were insufficient for NGS (Cases 2, 8, and 11).

Samples of, altogether, 24 patients (male/female ratio 13/11) were included in this prospective study. The average age was 57 years, ranging from 31 to 87 years. In total, 45 samples were analyzed, as 21 patients had matched tissue and LB specimens, while in three cases only LB was studied because of insufficient tdRNA yield of tissue needle aspiration (Cases 2, 8, and 11). The study workflow is illustrated in a flow chart (Figure 1).

### 3.2. Histological Features Including Immunohistochemistry and FISH

**Figure 2:**
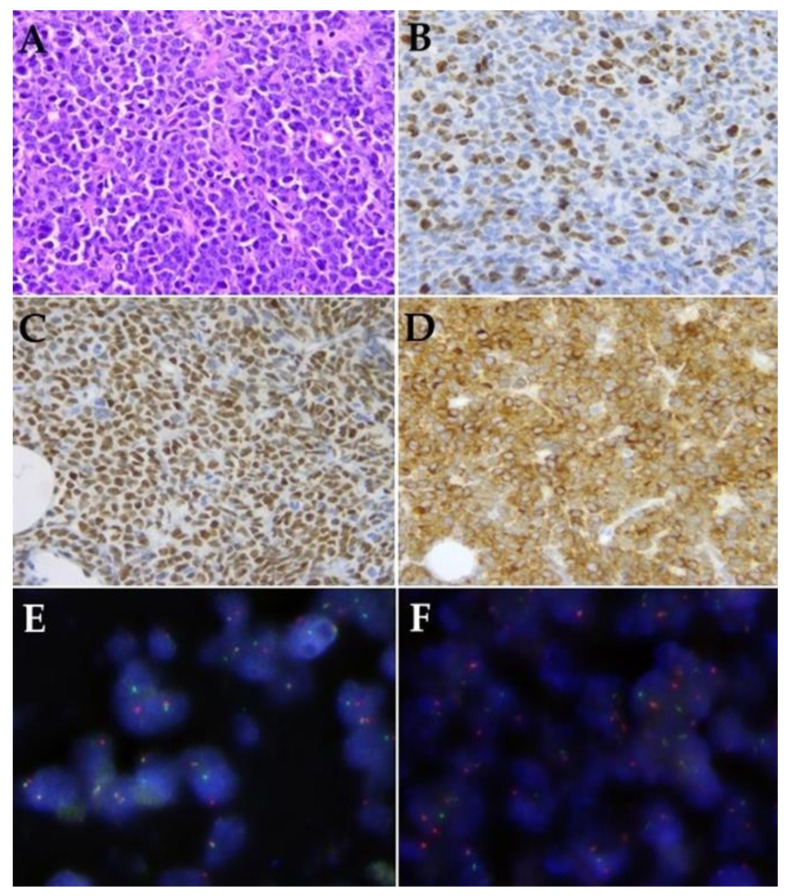
Histological (40×), immunohistochemical (IHC) (40×), and FISH (100x) features one of the double-hit cases (Case 18). (**A**): Conventional histological (H&E) record. (**B**): Ki-67 IHC. (**C**): *MYC* IHC. (**D**): *BCL2* IHC. (**E**): *MYC* FISH. (**F**): *BCL2* FISH.

Histopathological, IHC, and FISH features are summarized in Table 1. DLBCL with non-nodal origin was overrepresented in our series (12 cases), including lung and central nervous system manifestations. The cell-of-origin classification resulted in 4 germinal center B cell-like (GCB) and 15 non-GCB phenotypes. Two double-hit high-grade B-cell lymphoma cases were also included, featured by simultaneous *MYC* and *BCL2* translocations that were verified using FISH (Case 18, 19). H&E-stained slides, *MYC*, *BCL2* IHC, and FISH record one of the double-hit cases, which (Case 18) is presented in Figure 2. Further, two cases with *BCL2* and three cases with *MYC* alterations were included (NGS only detected *MYC* translocation in Case 3).

### 3.3. cfTNA Concentrations and Pre-Seq QC Assay

The processing of the LB samples resulted in good nucleic acid qualities with a mean cfDNA concentration of 9 ng/mL plasma (range: 1.5–24.6) and mean total cfRNA concentration of 541.8 pg/mL plasma with high yield variability (range: 3.75–1836).

Samples with Seq QC higher than 31 Cq failed. Both fusion transcripts and gene variants detected in tdRNA and matched cfTNA were carefully evaluated and compared.

### 3.4. Gene Fusions Detected by NGS

**Figure 3:**
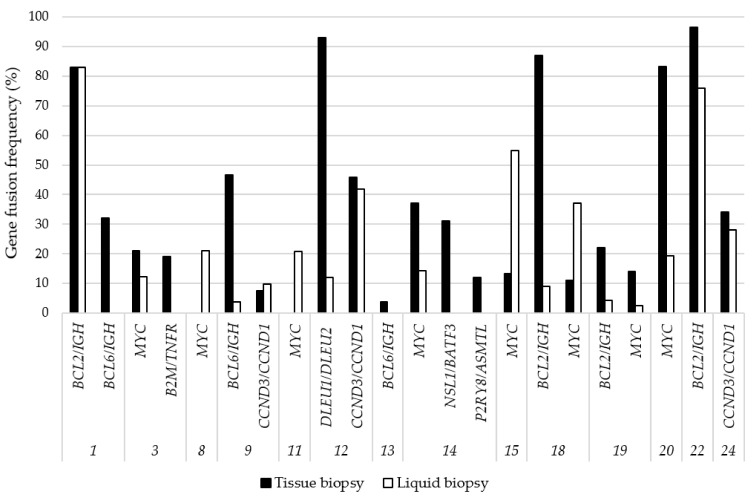
Gene fusions detected by NGS in matched samples originating from tissue biopsy and liquid biopsy. Case numbers refer to the study cohort displayed in Table 1. Gene fusion frequency was calculated for fusion transcript reads and the total reads ratio. In cases with *MYC* translocation, the applied NGS panel could not exactly distinguish immunoglobulin genes; therefore, the fusion partner (encoding one of the Ig chains) was not given.

Gene fusions identified throughout the tissue biopsy-derived tdRNA were generally in good agreement with the results obtained from the matched LB-derived cfTNA samples (Figure 3). Detected gene fusions are presented in Table 1. *BCL2*/*IGH* translocations were detected in all four LB samples derived from FISH and tissue NGS positive cases, and, similarly, the *MYC* translocation was identified in both sample types in six cases. In a further two cases, *MYC* fusions could be demonstrated from the cfTNA samples, while tissue biopsies failed due to technical reasons (Case 8, 11). A classical *BCL6*/*IGH* fusion was identified from another cfTNA in Case 9. Other gene fusions, such as *CCND3*/*CCND1* and *DLEU1*/*DLEU2*, were recovered with 100% concordance from plasma-derived cfTNA. The translocations *B2M*/*TNFR* (Case 3), *NSL1*/*BATF3*, and *P2RY8*/*ASMTL* (both in Case 14) were not represented in the plasma at the time of the sampling, and the frequency of these alterations in tissue was 19%, 31%, and 12%, respectively.

### 3.5. NGS-Based Mutation Profiling

In parallel with gene fusion detection, the RNA-based technology allowed us to capture single nucleotide variants (SNVs) from the same samples (Table 2). SNVs were identified in 23/24 patients (95.8%), and only one case (Case 24) remained free of nucleotide aberration by our method. In three cases, the LB sample was the only informative source for genetic analysis (tissue biopsy insufficient for molecular analysis, Case 2, 8, and 11).

The cfRNA variant allele frequencies (VAF) of tissue biopsy and LB were highly variable with a mean of 40.0% (range: 2.0–88.8) and 24.6% (range: 2.0–96.0), respectively. Pathogenic mutations were detected at a rate of 16/24 (66.7%). Pathogenic variants were obvious in some of the most commonly affected genes in lymphomas, such as *BCL2*, *MYD88*, *NOTCH2*, *EZH2*, and *CD79B*, and most of them could be identified in matched LB-originated cfRNA. Some of the SNVs found in tdRNA were not found in cfRNA, and in reverse, some gene variants were detected only in cfRNA (Cases 13, 20, and 23).

The number, type, and allele frequencies of SNVs detected were variable. In general, two or three nucleotide changes were provided. The highest number of nucleotide aberrations demonstrated was 5 (Case 15), while only one case remained negative for SNVs (Case 24). Variants were further categorized according to the clinical significance defined by the COSMIC database. Pathogenic SNVs were referred to as mutations (*n* = 22 in the 24 patients), while benign alterations were considered neutral (*n* = 5). Uncertain nucleotide changes (*n* = 34) were also frequently found. Exact genotypes (SNVs), VAF, and clinical significance from tissue biopsy and matched liquid biopsy samples are compared in Table 2.

## 4. Discussion

Due to the continuous diversification of lymphoma classes and the growing therapeutic opportunities, precise pathological and molecular testing is required. Lymphoma genotyping is currently mainly possible following invasive tissue biopsy sampling. However, tissue procurement and subtyping might be complicated or inconclusive due to limitations in sample size, partial involvement, or anatomically difficult sites. Moreover, repeated sampling is increasingly required to follow signs of progression.

In the area of precision oncology, therapies based on molecular genetic findings are increasingly applied. The common NGS platforms usually refer to the detection of SNVs and small insertions and deletions (indels) [16,17]. However, the efficient analysis of larger indels and structural variants such as gene fusions is also evolving. Recurring translocations are disease-specific and the identification of gene fusions is an increasingly important component also in lymphoma diagnostics [18]. Chromosomal breakpoints or aberrant protein expression may be covered by diverse, widely used clinical approaches, including fluorescence in situ hybridization (FISH), immunohistochemistry (IHC), or reverse-transcription PCR (RT-PCR). Neither FISH nor IHC provides fusion partner breakpoint precision and RT-PCR also requires knowledge on potential fusion partners.

The urgent clinical need motivated the release of fast and concentrated gene rearrangement NGS assays. Different platforms including target enrichment for NGS have been published and revised for this purpose [19]. One of the methods of target enrichment is hybridization capture, which elucidates the high versatility of hundreds of genes to the entire human genome [20], requiring long hybridization times, high yield of starting nucleic acids, and specialized design, synthesis, and optimization. A major disadvantage of this method is the lack of unique sequencing start sites, which may result in systematic errors at multiple levels [10].

The ratio of cell-free nucleic acid may be extremely low in the PB-derived plasma sample, which is a unique challenge for application workflows and analysis tools, especially for gene fusion detection. A sufficient read of coverage is essential to detect structural variations in PB plasma.

Several bioinformatics approaches were reported to identify gene rearrangements following sequencing, using disconcordant reads and/or split reads. Existing tools such as Breakdancer [21] use disconcordant mappings, while others such as Socrates [22] and SViCT [23] use split reads, as well. The latter software combines these two approaches. The effectiveness of cell-free nucleic acid mappings was evaluated in detail for solid tumors [23] but not in lymphomas so far. In our study, we primarily used a commercially available analysis software (Archer version 6.2) tool for fusion detection in lymphoma cfRNA.

According to our experience, the AMP target enrichment platform appeared to be a fast and effective way to simultaneously detect gene translocations and nucleotide variants [10,11]. This method guarantees increased confidence not only by determining the gene fusions but also by confirming that the fusion is recognized in transcribed mRNA. We have demonstrated its real-life utility for the detection of gene translocations and point mutations from low amounts of FFPE-derived tdRNA and also plasma-derived cfTNA samples. Genetic subtypes of aggressive lymphomas with distinct genotypic characteristics could be identified in a generally good agreement with lymphoma tissue-based results. Moreover, plasma LB genotyping also allowed for the recovery of fused genes and/or nucleotide variants which were suppressed in the biopsy sample for any reason. Further to technical problems at the tissue level, spatial tumor heterogeneity may significantly contribute to differences in the genetic profile seen in cfTNA samples [3]. In reverse, plasma cfTNA may underrepresent focal aberrations due to the limited release or to minor subclones.

The pathogenic aberrations detected in this series of cases could generally be associated with aggressive phenotype and poor prognosis. According to the COSMIC database, the *STAT6* pathogenic mutations (c.1249A > T; p.Asn417Tyr in cases 11, 15 and c.1256A > G; p.Asp419Gly in cases 1, 5, 21) correlate with DLBCL and FL progression, as well. Aberrations in *PAICS*, responsible for an enzyme involved in nucleotide biosynthesis were explored in correlation with poor prognosis in DLBCL patients [24]. In our study, DLBCL and one of the FL grade 3A cases showed up with *PAICS* alterations, which were considered to be SNP after comparison with the NCBI dsSNP database. Mutations of *CD79B* (Case 1) encoding the B lymphocyte antigen receptor Ig-β component and of *MYD88* (Case 6, 16, and 17) are well-known alterations in B-lymphoid malignancies, including PCNSL and leg-type cutaneous DLBCL. Another significant gene is *EZH2* (Case 12, 19, and 22), which participates in histone methylation and transcriptional repression and which gained interest as an important therapeutic target in FL [25]. The proto-oncogene serine/threonine-protein kinase *PIM1* (Case 16 and 17) and transmembrane protein *NOTCH2* (Case 8) gene aberrations are also characteristic for DLBCL (COSMIC). *XPO1* (encoding exportin protein) involvement (Case 16) was demonstrated in primary mediastinal B-cell lymphoma (PMBL) and classical Hodgkin lymphoma (cHL) [26]. On the contrary, the mutation of the *JAK2* gene best known as a driver in myeloproliferative neoplasias [27] presented with the alteration c.1177C > G; p.Leu393Val (Case 15) and was rather considered non-pathogenic SNP according to NCBI dsSNP search results.

In the PTCL case, only a *CCND3/CCND1* fusion was detected and no SNVs were found, although the most frequent PTCL-related genes (e.g., *DNMT3A*, and *IDH2* as well as a new highly prevalent *RHOA*) were all covered by our NGS panel.

Representative PB samples are of special value in lymphomas developing at critical anatomical localization, e.g., primary CNS lymphoma. In the present series, we were able to demonstrate lymphoma-related translocations and SNVs from the same LB samples of PCNSL patients (cases 15–17). Mutation of the *MYD88* gene has been reported in extranodal DLBCL with a high frequency, including PCNSL and leg-type cutaneous DLBCL (Case 6, 16, and 17). Although these results appear to be promising, we also stated that the yields of cfTNA isolated from the plasma of these patients were generally low (mean cfDNA concentration: 1.5 ng/mL plasma–range: 1.1–1.9, and mean total cfRNA concentration: 204 pg/mL plasma–range: 6.18–330). Future large-scale evaluation of LB results is required to demonstrate the exact clinical utility of the method for PCNS-DLBCL diagnostics.

## 5. Conclusions

Our prospective study demonstrates a novel non-invasive approach to analyze frequent gene fusions and variants in aggressive lymphomas in one session. Moreover, the approach served with new information in addition to the tissue-derived NGS data and reflected an extended landscape of gene aberrations. Standardized clinical applications using cell-free nucleic acids potentially reflect the spatial tumor heterogeneity and provide novel aspects for the precision treatment of aggressive lymphomas. PB LB sampling may substantially support the diagnostics of processes at anatomically critical sites (such as the CNS) at minimal procedural risks.

## Figures and Tables

**Table 1:** Histopathological, immunohistochemical (IHC), fluorescence in situ hybridization (FISH), and NGS gene fusion results of aggressive lymphoma patients. CNS: central nervous system, DLBCL: diffuse large B-cell lymphoma, PTCL: peripheral T-cell lymphoma, COO: cell of origin, GCB: germinal center B cell-like.

Case	Sex	Age (Year)	Localization	Histological Diagnosis	Tumor Ratio (%)	COO	IHC (%)			FISH (%)			Gene Fusions Detected by NGS
							Ki-67	MYC	BCL2	*MYC*	*BCL2*	*BCL6*	
1	F	50	lymph node	DLBCL	30	GCB	70	10	80	negative	54	25	*BCL2/IGH, BCL6/IGH*
2	M	87	lymph node	DLBCL	50	non-GCB	90	40	50	negative	negative	negative	negative
3	M	58	lymph node	DLBCL	70	non-GCB	70	20	10	negative	negative	negative	*MYC, B2M/TNFR*
4	M	62	lymph node	DLBCL	60	GCB	65	5	5	negative	negative	negative	negative
5	F	37	lymph node	DLBCL	90	non-GCB	40	20	15	negative	negative	negative	negative
6	M	73	skin	cutaneous DLBCL (leg type)	80	non-GCB	80	0	70	negative	negative	negative	negative
7	M	83	colon	DLBCL	60	non-GCB	50	5	100	negative	negative	negative	negative
8	F	68	liver	DLBCL	50	non-GCB	80	30	100	negative	negative	negative	*MYC*
9	F	67	liver	DLBCL	80	non-GCB	70	20	95	negative	negative	40	*BCL6/IGH, CCND3/CCND1*
10	F	58	parotis	DLBCL	60	non-GCB	30	0	40	negative	negative	negative	negative
11	F	58	lung	DLBCL	70	non-GCB	75	25	50	negative	negative	negative	*MYC*
12	M	45	lung	DLBCL	60	non-GCB	30	40	0	negative	negative	negative	*DLEU1/DLEU2*, *CCND3/CCND1*
13	M	47	lung	DLBCL	70	non-GCB	30	10	10	negative	negative	10	*BCL6/IGH*
14	M	72	kidney	DLBCL	60	non-GCB	90	60	60	88	negative	negative	*MYC*, *NSL1/BATF3*, *P2RY8/ASMTL*
15	F	31	CNS	DLBCL	50	non-GCB	70	15	10	5	negative	negative	*MYC*
16	F	64	CNS	DLBCL	80	non-GCB	80	30	70	negative	negative	negative	negative
17	F	51	CNS	DLBCL	80	non-GCB	90	20	80	negative	negative	negative	negative
18	M	47	lymph node	double hit DLBCL, high-grade	90	GCB	50	80	100	64	70	negative	*MYC, BCL2/IGH*
19	F	47	lymph node	double hit DLBCL, high-grade	80	GCB	90	80	90	74	80	negative	*MYC, BCL2/IGH*
20	M	53	upper lip	Burkitt	70	-	90	90	5	70	negative	negative	*MYC*
21	F	85	lymph node	follicular, grade 3A	50	-	25	0	30	negative	negative	negative	negative
22	M	42	lymph node	follicular, grade 3A	60	-	10	0	100	negative	80	negative	*BCL2/IGH*
23	M	50	parotis	follicular, grade 3A	70	-	15	5	100	negative	negative	negative	negative
24	M	37	lymph node	PTCL, high-grade	60	-	80	5	0	negative	negative	negative	*CCND3/CCND1*

**Table 2:** Detected gene variants. NGS on samples originating from tissue biopsy and matched liquid biopsy was performed. The number represents the case ID. VAF: variant allele frequency, SNP: single nucleotide polymorphism.

Case	Histological Diagnosis	Tumor Ratio (%)	Gene	Nucleotide Variant	Amino Acid Change	Tissue Biopsy VAF (%)	Liquid Biopsy VAF (%)	Clinical Significance
1	DLBCL	30	*CD79B*	c.573_575del	p.Glu192del	11.6	0	pathogenic
			*STAT6*	c.1256A > G	p.Asp419Gly	29.8	0	pathogenic
2	DLBCL	50	*RANBP1*	c.254A > G	p.His85Arg	insufficient for NGS	36	uncertain
3	DLBCL	70	*CCND3*	c.65G > A	p.Arg22His	8	0	uncertain
4	DLBCL	60	*PAICS*	c.422C > G	p.Ser141Cys	52	51	SNP
			*TNFRSF13B*	c.215G > A	p.Arg72His	42	54	likely benign
5	DLBCL	90	*ETV6*	c.838A > G	p.Asn280Asp	23	0	uncertain
			*RAB29*	c.-130-4G > A	splice region	44	49	uncertain
			*STAT6*	c.1256A > G	p.Asp419Gly	49	35	pathogenic
6	cutaneous DLBCL (leg type)	80	*MYD88*	c.794T > C	p.Leu265Pro	36.9	2.3	pathogenic
7	DLBCL	60	*CDKN2A*	c.442G > A	p.Ala148Thr	56	50	benign
			*CYB5R2*	c.488T > G	p.Leu163Trp	42	46.4	SNP
			*PAICS*	c.422C > G	p.Ser141Cys	47.9	49.5	SNP
8	DLBCL	50	*CYB5R2*	c.488T > G	p.Leu163Trp	insufficient for NGS	25.2	SNP
			*NOTCH2*	c.7198C > T	p.Arg2400Ter		27.6	pathogenic
			*PIM1*	c.322T > C	p.Cys108Arg		16	uncertain
9	DLBCL	80	*CD79B*	c.489G > A	p.Met163Ile	48	2.2	uncertain
			*ETV6*	c.26G > C	p.Ser9Thr	81.2	20	uncertain
			*PAICS*	c.422C > G	p.Ser141Cys	39.4	48.4	SNP
			*PIM1*	c.302C > A	p.Ala101Asp	28.2	6.6	uncertain
10	DLBCL	60	*CCND3*	c.71A > G	p.Glu24Gly	7.14	3	uncertain
			*RANBP1*	c.254A > G	p.His85Arg	2	36	uncertain
11	DLBCL	70	*EIF4A1*	c.6del	p.Ala3ArgfsTer35	insufficient for NGS	96	uncertain
			*NFKB2*	c.1947G > T	p.Leu649Phe		6.9	uncertain
			*PAICS*	c.1076T > C	p.Val359Ala		9.9	SNP
			*STAT6*	c.1249A > T	p.Asn417Tyr		28.5	pathogenic
12	DLBCL	60	*EZH2*	c.1921T > A	p.Tyr641Asn	46	19	pathogenic
			*TCF3*	c.1291_1293delinsAGT	p.Gly431Ser	46	50	SNP
13	DLBCL	70	*NFKB2*	c.1947G > T	p.Leu649Phe	0	5.3	uncertain
14	DLBCL	60	*TCF3*	c.1291_1293delinsAGT	p.Gly431Ser	64	53	SNP
15	DLBCL	50	*CCND3*	c.531_532delinsTG	p.Ser178Ala	83.3	52.1	uncertain
			*JAK2*	c.1177C > G	p.Leu393Val	74.5	75	SNP
			*PLCG2*	c.2011A > G	p.Ile671Val	33.4	35.4	benign
			*RANBP1*	c.254A > G	p.His85Arg	17.3	19.2	uncertain
			*STAT6*	c.1249A > T	p.Asn417Tyr	34.6	32.5	pathogenic
16	DLBCL	80	*LMO2*	c.35C > T	p.Pro12Leu	52	48	uncertain
			*MYD88*	c.794T > C	p.Leu265Pro	38	2	pathogenic
			*PIM1*	c.816G > C	p.Glu272Asp	44	0	pathogenic
			*XPO1*	c.1711G > A	p.Glu571Lys	40	0	pathogenic
17	DLBCL	80	*CCDC50*	c.363A > T	p.Leu121Phe	64	51.5	benign
			*MYD88*	c.794T > C	p.Leu265Pro	51.6	2	pathogenic
			*PIM1*	c.850C > T	p.Leu284Phe	88.8	2.2	pathogenic
			*PTPN1*	c.899G > A	p.Arg300Gln	49.1	0	uncertain
18	double-hit DLBCL, high-grade	90	*BCL2*	c.-287 + 8C > G	splice region	87.3	9.3	uncertain
			*BCR*	c.1461_1461 + 1insA	p.Ser488LysfsTer2	59.5	0	uncertain
			*KMT2A*	c.11321 + 2del	splice region	63.8	0	uncertain
			*RAB29*	c.-130-4G > A	splice region	24.8	0	uncertain
19	double-hit DLBCL, high-grade	80	*EIF4A1*	c.115C > T	p.Leu39Phe	10.7	0	uncertain
			*EZH2*	c.1922A > T	p.Tyr641Phe	56.9	32.5	pathogenic
			*TCF3*	c.1291_1293delinsAGT	p.Gly431Ser	37	52.3	SNP
20	Burkitt	70	*CCDC50*	c.363A > T	p.Leu121Phe	36	65	benign
			*CYB5R2*	c.488T > G	p.Leu163Trp	0	6	SNP
21	follicular, grade 3A	50	*CYB5R2*	c.488T > G	p.Leu163Trp	19.8	37	SNP
			*STAT6*	c.1256A > G	p.Asp419Gly	24.2	5.2	pathogenic
22	follicular, grade 3A	60	*BCL2*	c.-289C > T	splice region	84	8	pathogenic
			*CYB5R2*	c.488T > G	p.Leu163Trp	21	40	SNP
			*EZH2*	c.1922A > T	p.Tyr641Phe	45	10	pathogenic
			*MYD88*	c.664 + 2T > A	splice region	19	12	uncertain
23	follicular, grade 3A	70	*PAICS*	c.422C > G	p.Ser141Cys	23	50	SNP
			*RANBP1*	c.254A > G	p.His85Arg	0	29	uncertain
			*STAT6*	c.1263T > G	p.Asn421Lys	34	0	uncertain
24	PTCL, high-grade	60	*negative*	negative	negative	negative	negative	negative

## Data Availability

The data presented in this study are available on request from the corresponding author. The data are not publicly available to protect the rights of patients.
